# ATG4B is required for mTORC1‐mediated anabolic activity and is associated with clinical outcomes in non‐small cell lung cancer

**DOI:** 10.1002/2211-5463.70138

**Published:** 2025-10-09

**Authors:** Patrick J. Ryan, Bethany C. Guerra, Selina Uranga, Jessica M. Cardin, Steven E. Riechman, Mariana Janini Gomes, James D. Fluckey

**Affiliations:** ^1^ Muscle Biology Laboratory, Department of Kinesiology and Sports Management Texas A&M University College Station TX USA

**Keywords:** autophagy, cell signaling, metabolism, mTORC1, non‐small cell lung cancer

## Abstract

The complex interplay of metabolic signaling networks is critical to the pathophysiology of lung cancer. The anabolic mTORC1 kinase and catabolic process of autophagy are key among these regulatory pathways. While their relationship has long been viewed as a matter of simple inhibition, with mTORC1 as a negative regulator of autophagy, new evidence suggests that this relationship may be more nuanced than previously described. Here, we demonstrate that an autophagy‐related, ATG4B, is required for mTORC1 activity and is associated with negative clinical outcomes in non‐small cell lung cancer (NSCLC). Targeting ATG4B *in vitro* suppresses cell proliferation, protein synthesis rates, and mTORC1 signaling in a cellular model of NSCLC. In contrast, overexpressing the ATG4B protease in healthy models of lung tissue increased mTORC1 kinase activity in healthy lung cell models, indicating that an increase in ATG4B is sufficient to drive cellular anabolic signaling. Finally, we found that ATG4B expression is high in NSCLC patient tumors, is elevated in early‐stage cancer, and predicts survival in lung adenocarcinoma patients. Taken together, our results demonstrate that ATG4B is required for anabolic behavior in NSCLC, indicating that the autophagic cascade may be a required input for mTORC1 activity and cellular anabolism in lung cancer. These results have implications for the field of cancer biology more broadly, as they indicate that the far from being a simple target of mTORC1, the autophagic cascade may serve as a requisite input for anabolic signaling, casting new light on the relationship between these processes in cancer pathophysiology. [Correction added on 25 February 2026, after first online publication: 9th sentence has been revised as “Targeting ATG4B *in vitro* suppresses cell proliferation, protein synthesis rates, and mTORC1 signaling in a cellular model of NSCLC”].

## Graphical abstract

The relationship between anabolic and catabolic processes governing lung cancer cell growth is nuanced. We show that ATG4B, an autophagy regulator, is elevated in lung cancer and that high ATG4B is associated with worse patient outcomes. Targeting ATG4B in cells reduces growth, protein synthesis, and mTORC1 activity, demonstrating a new relationship between autophagy regulation and anabolic behavior in lung cancer.

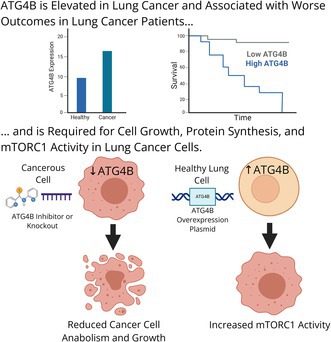

**Abbreviations**
4EBP1: eukaryotic initiation factor 4E‐binding protein 1
AMPK: AMP‐activated protein kinase
ATG4B: autophgay‐related 4B cysteine peptidase
LC3: microtubule‐associated protein 1A/1B light chain 3
LUAD: lung adenocarcinoma
LUSC: lung squamous cell carcinoma
mTOR: mechanisitc target of rapamycin
mTORC1: mechanisitc target of rapamycin, complex 1
NSCLC: non‐small cell lung cancer
P70S6K: ribosomal protein S6 kinase beta‐1

The third most common malignancy in the United States, lung cancer is also the deadliest tumor type in both men and women, claiming approximately 125 000 lives annually. Non‐small cell lung cancer (NSCLC) is by far the most common type of lung malignancy and is largely composed of two subtypes, lung adenocarcinoma (LUAD) and lung squamous cell carcinoma (LUSC). As with any malignant tumor, rampant and unregulated growth is a key hallmark of both types of NSCLC. At the heart of the complex metabolic machinery of the cancerous cell is the mechanistic target of rapamycin (mTOR) [1], a serine/threonine protein kinase responsible for regulating a broad range of cellular processes. The mTOR kinase forms two distinct complexes based on its associated binding partners: complex 1 (mTORC1), largely responsible for promoting cellular anabolism through its downstream effectors P70S6K and 4EBP1, and complex 2 (mTORC2), which has a less clearly defined role but may contribute to maintaining the actin cytoskeleton. Both mTOR complexes are key players in cancer cell biology; however, mTORC1 is the better studied and has been identified as a key driver of tumor pathology, including growth, metastasis, and immune escape in lung cancer [2, 3, 4].

Among its many complex interactions with other cell signaling mechanisms, one of the key functions of mTORC1 is to regulate the process of autophagy [5]. Autophagy (or macroautophagy) is a process by which a cell breaks down and destroys old, damaged, or abnormal proteins and other substances in its cytoplasm using a membrane‐bound autophagosome to deliver cargo to the lysosome for degradation. Much like the mTOR pathway, the contribution of the autophagic cascade to cancer pathology is complex and not fully elucidated. Autophagy is certainly critical to health and has largely been viewed as a response to cellular stress [6], supplying crucial metabolic substrate in the face of nutrient deprivation, for instance [7], or serving to clear the cell of misfolded proteins and dysfunctional organelles. In this capacity, autophagy plays a role in cancer prevention [8], protecting against the accumulation of cellular damage that may lead to the development of cancer. Indeed, the clearance of unneeded proteins from the cytoplasmic space is a key component of cellular proteostasis, the dynamic regulation of the cellular proteome [9]. However, while autophagy plays a protective role against cancer development, once a tumor is established, the function of autophagy shifts from tumor‐suppressing to tumor‐promoting, with cancers across various tissues of origin displaying a reliance upon autophagic proteolysis for continued cellular growth [10, 11]. Autophagy has accordingly been shown to be critical to chemotherapeutic drug resistance [12], metastasis [13], and immune escape [14] in NSCLC—all processes that involve a contribution from mTORC1.

The reasons for this shift to a pro‐pathological process remain unclear. A key player in regulating autophagy is mTORC1, which has a well‐documented role as an autophagic inhibitor, in addition to its established cancer‐promoting activities. One view of this relationship is that excess mTORC1 activity, by inhibiting autophagy, may limit the ability of the cell to clear accumulated proteins and prevent cellular damage and thus lead to cancer development. On the other hand, this view does not account for the reliance upon autophagy found in numerous cancers, coupled with mTORC1 hyperactivity. Indeed, studies have shown that inhibition of autophagy, in colon [15], breast [16], and prostate [16] cancers, also suppresses cellular functions associated with mTORC1 activity. Those results join others in noncancerous tissues showing that the relationship between autophagy and mTORC1‐mediated anabolic behavior may be more complex than a simple mechanism of unidirectional inhibition [17, 18, 19]. In light of the evidence provided in the literature, and our own prior work demonstrating that a selective autophagy inhibitor prevents anabolic behavior in healthy tissues [19], we here set out to investigate the role of autophagy inhibition on mTORC1 activity in lung cancer.

The autophagic cascade is a complex, multistage process, with numerous different regulatory steps and checkpoints. Various drugs have been designed to act on this process, from the inhibitor of autophagosomal–lysosomal fusion chloroquine and its derivatives to newer inhibitors of specific autophagy‐related enzymes. Among these newer compounds are an array of inhibitors targeting the cysteine protease ATG4B. ATG4B is involved in both the processing and delipidation of LC3, thus serving as a key step in autophagosomal formation [20]. While there are four ATG4 homologs (A–D), ATG4B has the highest processing activity toward LC3 [20, 21], and as such has been the target for several pharmacological inhibitors aimed at inhibiting the early stages of autophagy, with reports demonstrating the efficacy of therapeutics targeting ATG4B in breast [22], brain cancer [23], gastric [24], and colorectal [25] cancer. While less work has been done in determining the effect of ATG4B targeting in lung cancer, prior work has shown that ATG4B inhibitors potentiate the effect of cisplatin chemotherapy [26] in lung cancer cells, indicating that this enzyme makes some contribution to lung cancer pathology. Based on our previous work showing that autophagy is required for cellular anabolism, we hypothesized that ATG4B would be upregulated in NSCLC and that inhibition of this enzyme would suppress anabolism and combat pathology in lung cancer.

## Materials and methods

### Cell culture

To study the role of ATG4B in regulating cell biology, we employed the following, all purchased from ATCC (Manassas, VA, USA): A549 (a model of NSCLC, ATCC Cat# CCL‐185, RRID:CVCL_0023), and BEAS‐2B (a model of healthy bronchial epithelium, ATCC Cat# CRL‐3588, RRID:CVCL_0168). A549 cells were cultured in Dulbecco's modified essential media (DMEM; 11995040; Gibco, Grand Island, NY, USA) and BEAS‐2B cells in α‐MEM (45000‐300, Corning Incorporated). All media were supplemented with 10% fetal bovine serum (1500‐500; Avantor, Radnor, PA, USA) and 1% penicillin/streptomycin (Avantor), and cells were maintained in a humidified chamber maintained at 37 °C and 5% CO_2_. [Correction added on 25 February 2026, after first online publication: The above paragraph has been updated].

### ATG4B inhibition, knockdown, and overexpression

ATG4B targeting was achieved by either pharmacological inhibition or genetic knockdown. The small‐molecule ATG4B specific inhibitor NSC185058 was a generous gift from the laboratory group of William A. Dunn, who developed the compound, and was used at a dosage of 100 μm in all experiments (with an isomolar amount of DMSO serving as a control). For knockdown experiments, silencing RNA to ATG4B (4392420:s23244, ThermoFisher Scientific, Waltham, MA, USA) was used to target the ATG4B transcript for RNA interference, with negative control siRNA (4390843, ThermoFisher) serving as a control. Finally, to overexpress ATG4B in healthy cells, a custom ATG4B overexpression plasmid was built (OHu29105D Accession No: NM_013325.5, Vector: pcDNA3.1+/C‐(k)DYK, GenScript Biotech, Piscataway, NJ, USA) and transfected into cells using Lipofectamine 3000 per the manufacturer's instructions (L3000015, ThermoFisher), with an empty backbone plasmid (Vector: pcDNA3.1+/C‐(k)DYK, GenScript Biotech) as a control.

### Cell proliferation assays

Cellular proliferation was measured using a WST‐8 assay (HY‐K0301, MedChemExpress, Monmouth Junction, NJ, USA), a colorimetric assay that produces a tetrazolium salt in proportion to the number of cells in a sample well. For experiments involving pharmacological inhibition, cells were seeded in 96‐well plates at a density of 5000 cells per well and allowed to attach overnight. The next day, media from each well was aspirated, and cells were treated with either inhibitor or vehicle control at a concentration of 100 μm. From this point, every 24 h, the media from wells was again aspirated and refreshed with media containing 10% WST‐8 assay solution, per manufacturer's instructions. Plates were gently rocked, then returned to the incubator to allow the WST‐8 reaction to proceed for 1 h. Subsequently, the absorbance at 450 nm of each assayed well was measured using a plate reader. The process of incubation and reading was repeated at 48 and 72 h. For RNA interference experiments, cells were reverse transfected by adding cells at a density of 10 000 cells per well to transfection agents (prepared using the aforementioned siRNA adducts plus Lipofectamine RNAiMAX (13778150, ThermoFisher) mixed in Transfectagro transfection medium (40‐300‐CV, Corning)), containing ATG4B or control siRNA. Cells were transfected overnight with media replenished the following day. 24 h later, cell proliferation assays were conducted exactly as described for pharmacological inhibitor experiments.

### Protein synthesis measurements

Protein synthesis rates were measured using deuterium oxide incorporation as described in Gasier *et al*. [27], with some modifications. Similar to proliferation assays, pharmacological experiments were conducted by seeding cells at a density of 50 000 cells per well in 24‐well plates. The following day, cells were treated with media containing NSC185058 or a vehicle control, supplemented with 4% deuterium oxide for 24 h. For RNA interference, cells were again reverse transfected, replenished with their normal media the next day, then exposed to deuterium oxide for 24 h. Following the 24‐h tracer incorporation period, a sample of media from each well was retained, while cells were harvested in lysis buffer (containing 5 mm glycerophosphate, 20 μm ATP, 25 mm HEPES, 25 mm benzamidine, 2 mm PMSF, 4 mm EDTA, 10 mm magnesium chloride, 100 mm sodium fluoride, 10 mm sodium orthovanadate, supplemented with Halt protease and phosphatase inhibitor cocktail (78437, ThermoFisher)), sonicated for five 10 s cycles at a frequency of 40 kHz, then diluted to a final concentration of 10% trichloroacetic acid (TCA). To separate cellular protein from other components, samples were then centrifuged at 1600 ***g*** for 15 min, with the subsequent supernatant (containing free amino acids) discarded, and the pellet (containing cellular protein) retained. The pellet was resuspended in 10% TCA and centrifuged once more. Isolated proteins in the final pellet were hydrolyzed to constituent amino acids by the addition 6 N HCl overnight in a heating block set to 100 °C until samples were homogenous. Subsequently, samples were derivatized using a 3 : 2 : 1 ratio solution of methyl‐8, acetone, and acetonitrile, then loaded onto a gas‐chromatograph‐mass spectrometer, and the enrichment of cellular alanine (*E*_A_) was determined. Separately, cell media was thawed then incubated in a solution of 10 N NaOH in 5% (vol/vol) acetone:acetonitrile for 24 h, then mixed with *N*‐hexane and extracted for analysis. Enrichment of the media (*E*_CM_) was calculated using gas chromatography–mass spectrometry (GCMS, Agilent 7890a GC/5975c VL MSD, Santa Clara, CA, USA), and protein synthesis rate was calculated using the equation $\frac{E_{A}}{E_{\mathrm{CM}}\times 3.7\times t\left(h\right)\;}\times 100$, where *E*_A_ represents amount of protein‐bound [^2^H]alanine (mole% excess), *E*_CM_ is the quantity of ^2^H_2_O in cell media (mole% excess), 3.7 represents the exchange of ^2^H between cell media and alanine (e.g., 3.7 of 4 carbon‐bound hydrogen of alanine exchange with water), and *t*(*h*) is the duration of tracer exposure measured in hours.

### Western immunoblotting

To assess cellular protein content and signaling status, cells were grown and treated in 24‐well plates as described above, then harvested in lysis buffer. Cytosolic protein concentration was calculated using a BCA assay, and samples were mixed with 4× Laemmli buffer before being resolved on a polyacrylamide gel for 20 min at an open voltage fluctuating between 100 and 150 V, 40 mA, and 100 W, followed by 80 min at 80 mA and 100 W. All target proteins were separated on 12% polyacrylamide gels. Following SDS/PAGE, samples were transferred to a fortified nitrocellulose membrane (10120‐018, VWR, Radnor, PA, USA) using a semi‐dry transfer system for 55 min at an open voltage fluctuating between 10 and 15 V, 250 mA, and 4 W. Equal loading was verified by Ponceau S staining, and membranes were subsequently blocked in 5% milk for 1 h, washed three times in TBS, then incubated in primary antibody solution at a concentration of 1 : 1000 on a rocker in a 4 °C refrigerator overnight. Primary antibodies used were: ATG4B (Cell Signaling Technology, Cat# 13507, RRID:AB_2750642), Phosphorylated P70S6^Thr389^ Kinase (Cat# 9205 (also 9205L, 9205S), RRID:AB_330944), Total P70S6 Kinase (Cat# 9202 (also 9202L, 9202S), RRID:AB_331676), Phosphorylated 4EBP1^Thr37/46^ (Cat# 2855 (also 2855L, 2855S, 2855P), RRID:AB_560835), Total 4EBP1 (Cat# 9644 (also 9644S, 9644P), RRID:AB_2097841). After this period, membranes were washed three times in TBS, incubated in goat anti‐rabbit conjugated HRP secondary antibody (Cell Signaling Technology, Cat# 7074 (also 7074S, 7074V, 7074P2), RRID:AB_2099233) of concentrations from 1 : 5000 to 1 : 100 000 solution for 1 h, washed again three times in TBS, and finally incubated in SuperSignal West Pico PLUS chemiluminescent substrate (34578, ThermoFisher, Waltham, MA, USA) for 1 min. Detection of the indicated probes was achieved using a cytvia imagequant 800 biomolecular imager, and densitometric analysis was conducted using cytvia imagequant 400 software, with signal intensities of measured bands normalized to the intensity of whole‐lane Ponceau red staining.

### Bioinformatics analysis

The UALCAN web tool [28, 29] was used to compare ATG4B gene expression in LUAD (*n* = 515) and LUSC (*n* = 503) to normal tissues (*n* = 59 for LUAD comparison and *n* = 52 for LUSC comparison) in samples acquired from the Cancer Genome Atlas (TCGA). UALCAN also was used to explore ATG4B expression across gender, cancer stage, nodal metastasis status, p53 mutation type, race, and smoking history in 114 normal and 1097 primary tumors. Separately, the Kaplan–Meier Plotter tool [30] was used to conduct Cox regression, assessing overall (OS, LUAD: *n* = 1161, LUSC: *n* = 780) and progression‐free survival (FP, LUAD: *n* = 906, LUSC: *n* = 220) in both types of NSCLC, respectively. Cohorts were split through auto‐selected best cutoff criteria, allowing for calculation of false discovery rates (FDR) along with log‐rank *P*‐values.

### Statistical analysis

Statistically significant differences in protein synthesis and western immunoblot measures were established using a two‐tailed *t*‐test, while differences in proliferation measures were determined by a two‐way ANOVA (group × time) with the Sidak correction used in the case of significant differences. graphpad prism (GraphPad Software, Boston, MA, USA) software was used for the analysis of these experiments. Welch's *t*‐test was used to assess significance in ATG4B gene expression between indicated groups, while Cox regression was used to evaluate differences in survival between high and low ATG4B gene expression. Statistical analysis for these later experiments was performed using built‐in tools on their respective web servers. All α levels were set at 0.05.

## Results

### Targeting ATG4B slows cell proliferation in NSCLC

**Fig. 1:**
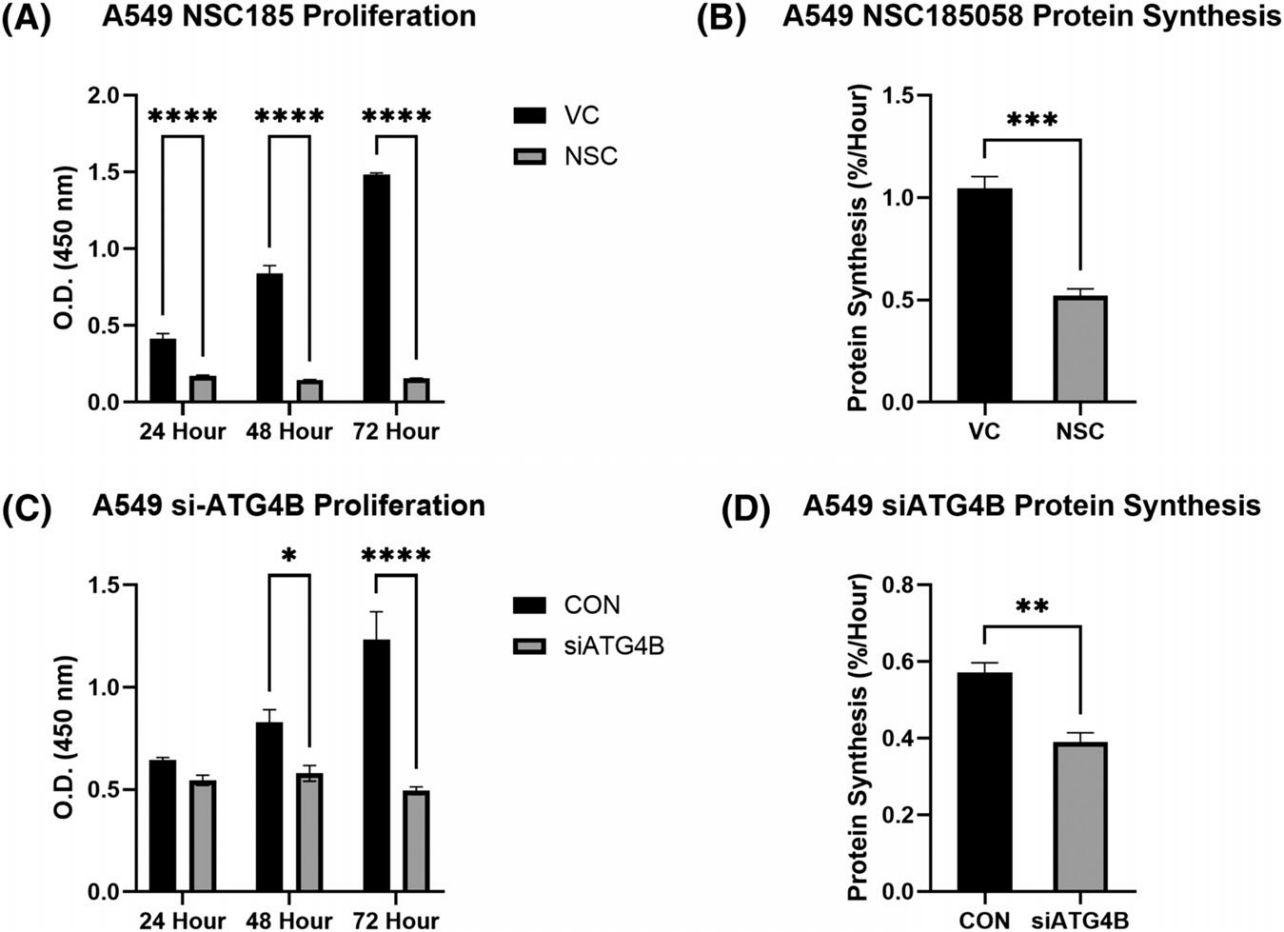
Targeting ATG4B suppresses cell growth and protein synthesis in NSCLC. Both pharmacological treatment (Panels A & B) and silencing RNA targeting ATG4B (Panels C & D) cause reductions in cell proliferation and protein synthesis rates in A549 NSCLC cells. * indicates a significant difference (*P* < 0.05), **(*P* < 0.01), ***(*P* < 0.001), ****(*P* < 0.0001), assessed by two‐way ANOVA with a Sidak correction in the case of significant differences. VC, vehicle control; NSC = NSC18505. Figures are presented as means with standard errors, all *n* = 4. [Correction added on 25 February 2026, after first online publication: Figure 1 caption has been updated].

Both NSC185058 (NSC), a small‐molecule inhibitor of ATG4B, and silencing RNA to ATG4B (siATG4B) significantly reduced ATG4B protein content in the A549 cell line (Fig. S1; A549 NSC: −49.3%; A549 siATG4B: −76.2%, *P* < 0.05) relative to respective controls. Accordingly, both treatments slowed cell proliferation across 3 days in A549 cells (Fig. 1; NSC: 24 h: −58.8%, 48 h: −82.6%, 72 h: −89.5%; siATG4B: 48 h: −30.0%, 72 h: −60.0%; all *P* < 0.05) indicating that the ATG4B protease is required for cell growth in NSCLC. These experiments were repeated with both control and experimental groups treated with Compound C (Dosomorphin), a small‐molecule inhibitor of AMPK, to test for the contribution of AMPK to the mechanistic action of NSC185058 and si‐ATG4B. As before, both treatments caused significant reductions in cell proliferation across 3 days, indicating that targeting ATG4B suppresses cell growth through an AMPK‐independent mechanism (Fig. S2). [Correction added on 25 February 2026, after first online publication: The above paragraph has been updated].

### ATG4B is required for mTORC1‐mediated protein anabolism in LUAD

**Fig. 2:**
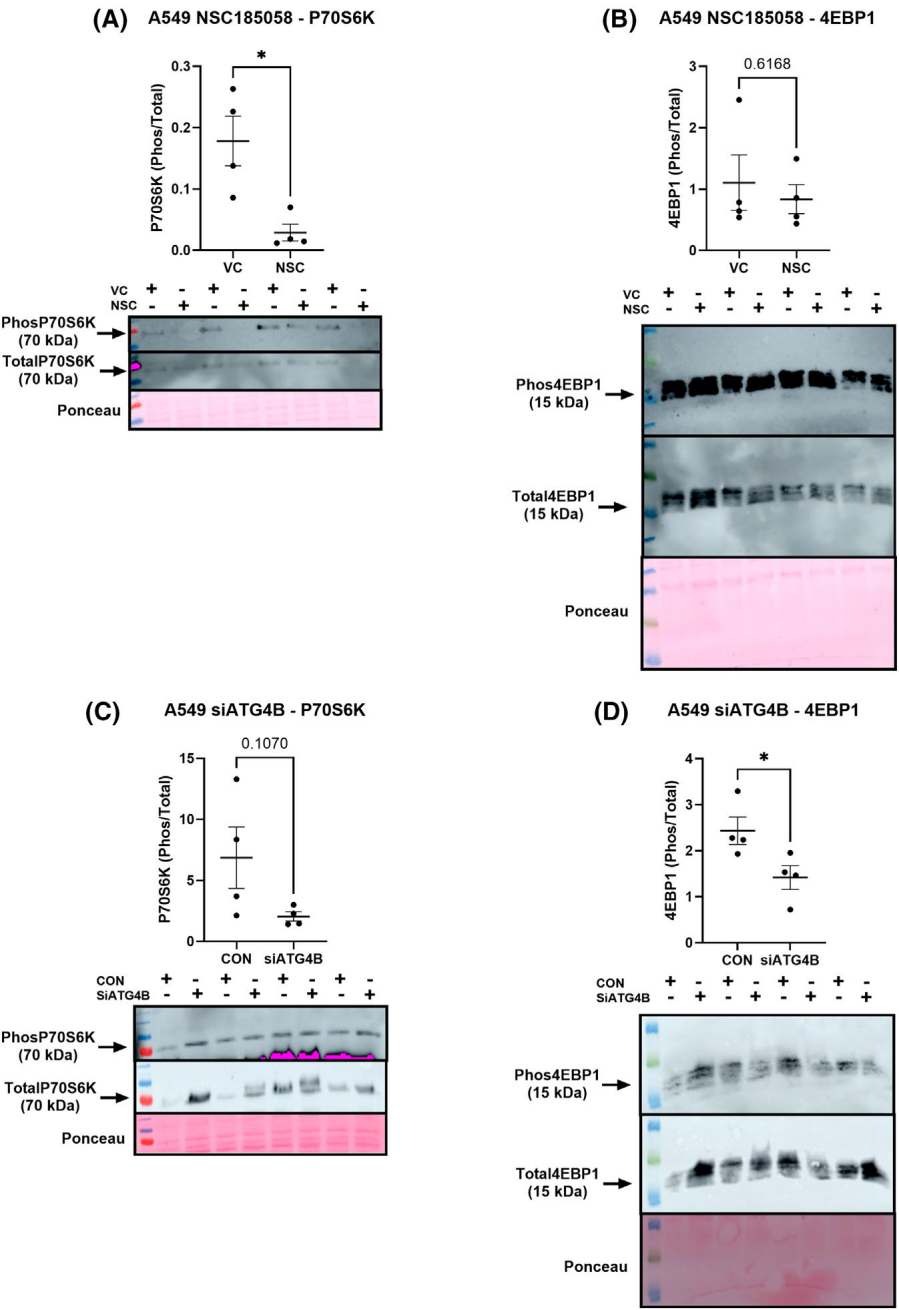
ATG4B is required for mTORC1 activity in cultured models of LUAD. The ATG4B inhibitor NSC185058 (panels A & B) and silencing RNA against ATG4B (panels C & D) suppress mTORC1 phosphorylation of P70S6K and 4EBP1 in lung adenocarcinoma cells (A549 cell line). * indicates a significant difference (*P* < 0.05), assessed by a two‐tailed *t*‐test. VC, vehicle control, NSC = NSC185058. Figures are presented as dot plots with means and standard errors, all *n* = 4.

**Fig. 3:**
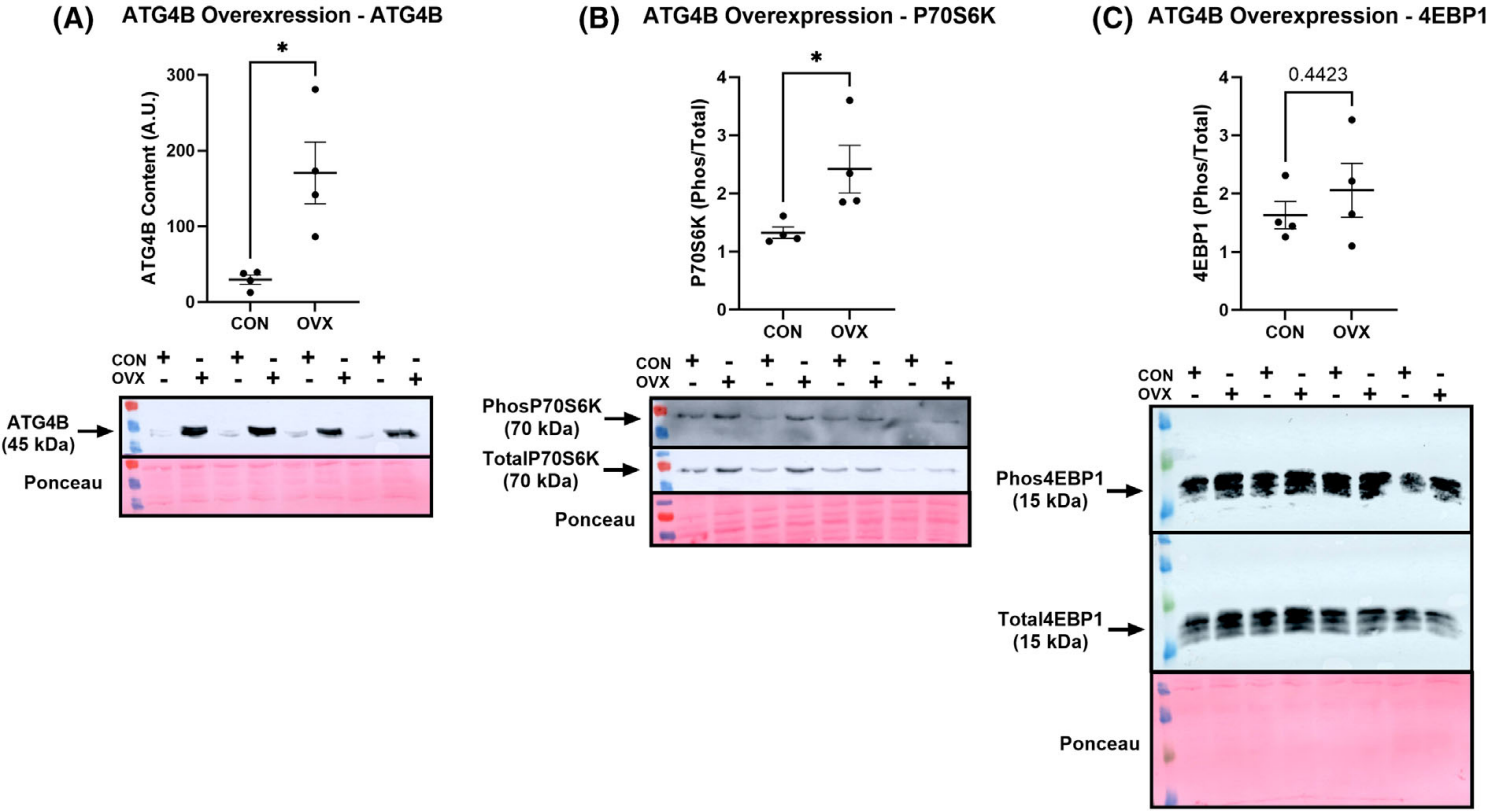
ATG4B overexpression promotes mTORC1 activity. Increases (OVX) in ATG4B protein content (panel A) cause an increase in the phosphorylation of P70S6K (but not 4EBP1) in a cultured model of healthy lung tissue (BEAS‐2B), indicating that ATG4B overexpression is sufficient to stimulate mTORC1 activity. * Indicates a significant difference (*P* < 0.05), assessed by a two‐tailed *t*‐test. Figures are presented as dot plots with means and standard errors, all *n* = 4.

NSC and siATG4B treatments resulted in reduced protein synthesis rates in A549 cells (Fig. 1; NSC: −50.2%; A549 siATG4B: −31.7%, *P* < 0.05). Both pharmacological and genetic targeting of ATG4B resulted in reductions in mTORC1 activity in A549 cells, although these effects differed modality (Fig. 2). NSC suppressed P70S6K phosphorylation by 83% (*P* < 0.05), with no significant effect on 4EBP1, while siATG4B resulted in a 41.7% decrease in 4EBP1 phosphorylation, with no effect on P70S6K (Fig. 2). Separately, to test the effect of an increase in ATG4B content on signaling in healthy cells, we conducted an overexpression experiment to increase ATG4B content in the BEAS‐2B cell line, a model of normal human bronchial epithelium (Fig. 3). We were successful in increasing ATG4B protein content, and while there was no difference in 4EBP1 measures, we did observe an 82% increase in P70S6K phosphorylation (Fig. 3, *P* < 0.05), indicating that increased expression of ATG4B alone is sufficient to drive mTORC1 activity. [Correction added on 25 February 2026, after first online publication: The above paragraph has been updated].

### ATG4B is overexpressed and associated with clinical factors in NSCLC

**Fig. 4:**
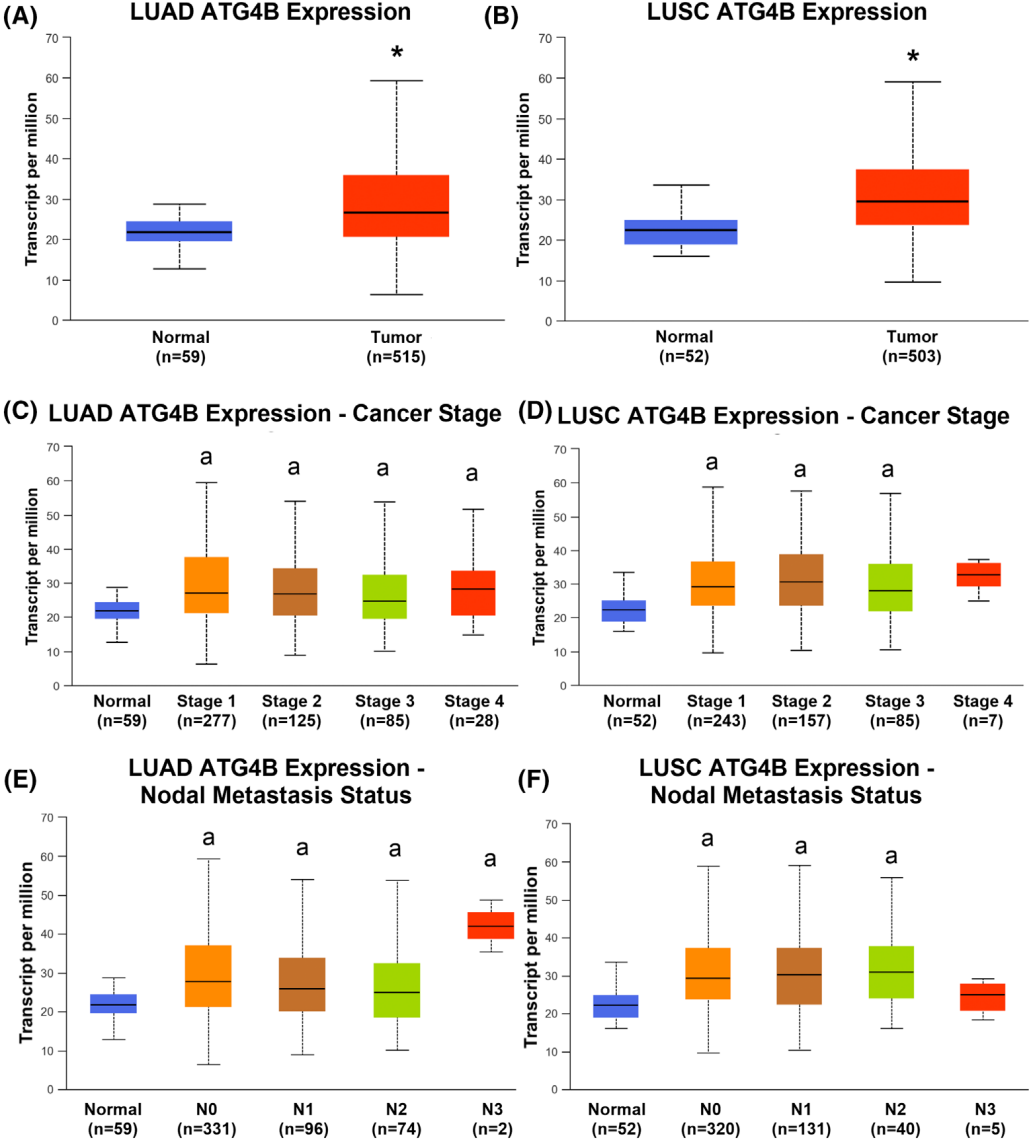
ATG4B is associated with cancer pathology in LUAD and LUSC. Expression of ATG4B transcript is higher in tumor tissues compared to normal ones (panels A & B) in both lung adenocarcinoma (LUAD) and squamous cell carcinoma (LUSC) and is high from an early‐stage (panels C & D). Further, ATG4B expression is high in localized tumors and remains high in metastatic ones (panels E & F). * Indicates a significant difference (*P* < 0.05), ‘a’ indicates a significant difference (*P* < 0.05) from normal tissues. Statistical significance was assessed by Welch's test. Figures are presented as box and whisker plots with respective *n* listed below each *x*‐axis.

ATG4B transcript levels were 21.8% higher in LUAD primary tumors compared to healthy controls (Fig. 4; 26.664 vs. 21.886, *P* < 0.05), and 31.9% higher in LUSC primary tumors compared to healthy control tissues (Fig. 4; 29.549 vs. 22.404, *P* < 0.05). Similarly, ATG4B transcript levels were significantly elevated in all stages of LUAD, at similar levels, compared to normal lung tissue (Fig. 4; *P* < 0.05) with no differences between stages, and in all but Stage 4 LUSC when compared to healthy controls (Fig. 4; *P* < 0.05, no differences between stages), indicating that increases in ATG4B expression occur early in the development of NSCLC. Similarly, ATG4B expression was high across all nodal metastasis (N) categories, with no differences between N status, in LUAD (Fig. 4). This trend holds for N status in LUSC as well, with the exception of N3, which was not statistically different from normal tissues, likely limited by low sample size in the N3 category (*n* = 5, Fig. 4). There were no differences observed between genders in either LUAD or LUSC (Fig. S3).

**Fig. 5:**
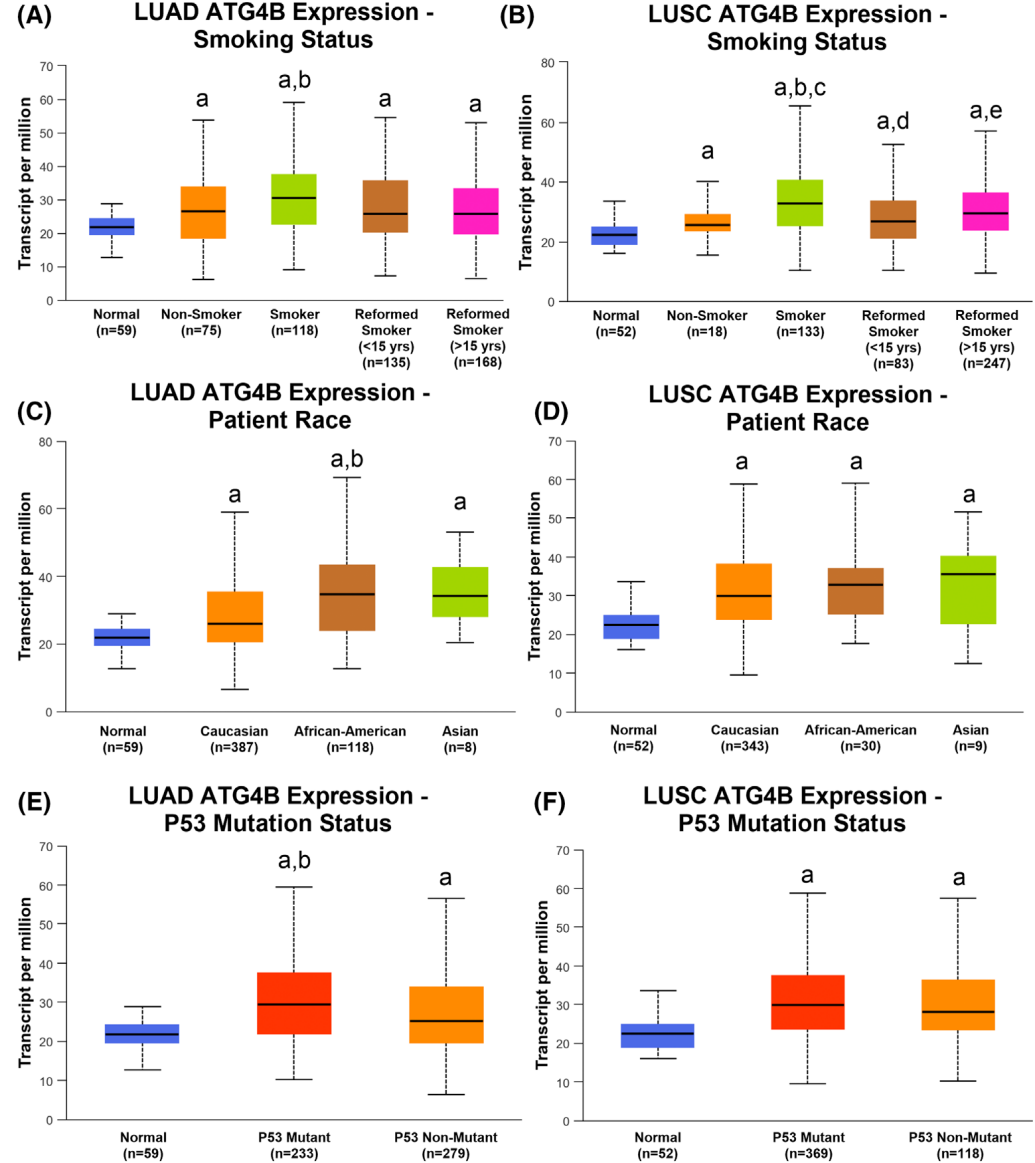
ATG4B and clinical factors in NSCLC. ATG4B expression is high in both smokers and nonsmokers compared to normal tissues in non‐small cell lung cancer (NSCLC). In lung adenocarcinoma (LUAD), ATG4B expression is higher in current smokers compared to reformed smokers (panel A), while levels are higher for both smokers compared to both nonsmokers and reformed smokers in lung squamous cell carcinoma (LUSC, panel B). On the other hand, ATG4B expression is higher in African‐American LUAD patients compared to Caucasian ones (panel C), while no differences based on race were found in LUSC (panel D). Finally, ATG4B expression is higher in LUAD tumors carrying a p53 mutation than those with wild‐type p53 (panel E), with no such difference found in LUSC (panel F). It is worth noting that ATG4B is higher in every type of NSCLC compared to normal tissues across characteristics. An ‘a’ indicates a significant difference (*P* < 0.05) from normal tissues, ‘b’ indicates a significant difference from nonsmokers, ‘c’ from both reformed smoker categories, ‘d’ from Caucasian race, and ‘e’ from nonmutant p53 tumors. Statistical significance was assessed by Welch's test. Figures are presented as box and whisker plots with respective n listed below each *x*‐axis.

However, some intriguing differences exist in ATG4B gene expression between LUAD and LUSC. In LUSC, ATG4B levels were higher in both smokers compared to nonsmokers and reformed smokers of less than 15 years, although differences between smokers of greater than 15 years did not rise to the level of statistical significance (Fig. 5). LUSC tumors, regardless of smoking status, were higher than normal healthy tissues. In contrast, ATG4B expression did not differ between smokers and nonsmokers in LUAD (Fig. 5) but was significantly lower in both categories of reformed smokers compared to current smokers. ATG4B transcript was high in LUAD tumors containing a mutation in the tumor‐suppressing p53 gene compared to both normal tissues and tumors without a p53 mutation (Fig. 5), with no differences between p53 mutants and nonmutants in LUSC (although both tumor types had higher ATG4B expression than normal tissues). Finally, while no difference in ATG4B transcript levels was observed between races in LUSC, its expression was significantly higher in African Americans compared to Caucasians in LUAD (Fig. 5).

### ATG4B predicts cancer mortality and progression in LUAD, but not LUSC

**Fig. 6:**
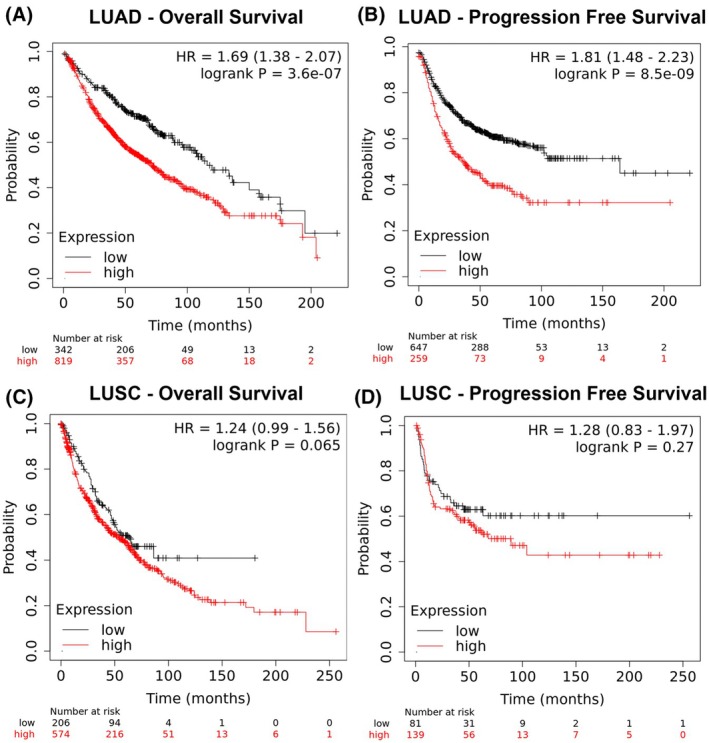
ATG4B expression predicts survival in LUAD, but not LUSC. High ATG4B transcript is associated with increased mortality (panel A) and progression‐free survival (panel B) in lung adenocarcinoma (LUAD). However, no effect was observed in lung squamous cell carcinoma (LUSC, panels C & D). Statistical significance was assessed by log‐rank test. HR, hazard ratio. Figures are presented as Kaplan–Meier plots with respective hazard ratios and *P*‐values in the upper right of each panel.

High ATG4B expression was associated with a hazard ratio of 1.69 (95% CI: 1.38–2.07) for overall mortality when compared to low ATG4B expression (*P* < 0.05), and a 1.81 (95% CI: 1.48–2.23) increase in hazard ratio for first progression (*P* < 0.05), such that median overall survival increased from 71 to 116 months in the high versus low ATG4B cohorts, and time to first progression increased from 37 to 164 months when comparing low versus high cohorts in LUAD (Fig. 6). On the other hand, neither overall survival (HR = 1.24 (95% CI: 0.99–1.56), *P* = 0.065), nor time to first progression (HR = 1.28 (95% CI: 0.83–1.97), *P* = 0.27) was statistically different between high and low cohorts in LUSC (Fig. 6).

## Discussion

The contribution of autophagy to lung cancer pathology is complex. While some investigations have indicated that autophagy plays a valuable role in protecting against cancer development [8], others have shown that the autophagic cascade is a critical contributor to numerous aspects of the cancerous phenotype, including tumorigenesis [31], progression [32], and drug resistance [33]. Our results support this latter view as we find that, in established tumors, the autophagic regulator ATG4B is an essential contributor to lung cancer pathology. We demonstrate ATG4B is required for cell growth and protein anabolism in cultured models of NSCLC and provide a novel insight into the contribution of autophagy to cancer cell signaling, providing evidence  that ATG4B is required for mTORC1‐mediated kinase activity in LUAD. Further, we show that an increase in ATG4B alone is sufficient to stimulate mTORC1 activity in noncancerous cells of the lung, indicating that ATG4B may be driving cancer pathology by activating the mTORC1 kinase. These findings have clinical relevance in lung cancer, as we report that ATG4B gene expression is greater in NSCLC tissues compared to healthy controls, is elevated in early stages of cancer development, and that high ATG4B levels were associated with increased risk of mortality in cancer progression in LUAD patients.

Unchecked anabolic signaling and rampant cell growth are key hallmarks of cancer pathology [34], with mTORC1 serving as a central hub to integrate various inputs (including from various oncogenic signals) and promote cell growth via increases in protein synthesis and progression through the cell cycle [35]. As such, mTORC1 is a critical mediator of lung malignancy, playing a role in virtually every pathological process underlying cancerous growth in the lung, including canonically acting as an inhibitor of autophagy. As with the contribution of autophagy to cancer growth, however, the relationship between the anabolic mTORC1 kinase and the catabolic process of the autophagic cascade is similarly complex. Traditionally, mTORC1 has been viewed as a unidirectional inhibitor of autophagy in cancer, with numerous studies documenting an increase in autophagic signaling with specific mTOR inhibitors [36, 37], or chemotherapeutic drugs such as cisplatin [38, 39]. On the other hand, there is evidence to suggest that this relationship is more nuanced than previously acknowledged. Other reports have demonstrated that autophagy not only contributes to cancer cell growth and drug resistance—processes involving mTORC1—but also that cisplatin‐induced autophagy increases phosphorylation of the core mTOR kinase in lung cancer [40]. In line with its role in cancer pathology, it appears that autophagy has a similarly multifaceted relationship with the mTOR pathway, functioning as either a target for inhibition or a required input of the mTORC1 kinase depending on the cellular context. Indeed, in previous work we demonstrated that a contribution from autophagy (mediated by ATG4B) is required for mTORC1 activity and cellular anabolism in skeletal muscle [19], proposing that autophagy is a required input for anabolism and the inhibition of autophagy by mTORC1 represents a negative feedback loop which intrinsically regulates cell growth. Based on our present findings, we argue that the overexpression of ATG4B and elevated autophagic activity in lung cancer represent a consequence of perturbation in this dynamic—that enhanced autophagy, in this case mediated by ATG4B, contributes to lung cancer pathology by promoting and sustaining mTORC1 activity.

We posit that these results represent a new understanding of cellular metabolism in lung cancer; specifically, that autophagy is a required input for mTORC1 and that increases in autophagic capacity may, in fact, promote cancer pathology by activating this central anabolic kinase. Our results, along with others, provide further evidence that autophagic catabolism is not merely a pathway for protein disposal, but a dynamic process of cellular adaptation that enables a broad range of cellular responses and reorganization. It stands to reason that malignant cells, with extraordinary requirements of sustained growth and cellular remodeling, would place high demand on this process. Our results show that targeting a specific autophagic checkpoint is sufficient to not only slow cell growth, but to suppress protein anabolism and mTORC1 activity, indicating that NSCLC cells are uniquely reliant upon ATG4B to sustain anabolic activity. While previous investigations have documented the contribution of ATG4B to cancer pathology in other tumor types, we are among the few to investigate the importance of this critical regulator in lung cancer, and the first to document that autophagy, mediated by ATG4B, is a directly required input for mTORC1‐mediated anabolism in any type of malignancy. These results hint at a new understanding of cancer cell biology:that the growth of cancerous cells is not simply regulated by anabolic kinases but is instead a coordinated behavior brought about by the integration of anabolic and catabolic signaling dynamics, in a process of integrated protein metabolism – demonstrating that catabolic processes are essential for anabolic ones in lung, and potentially other, cancers.

Lung cancer is a devastating disease, responsible for an estimated 125 000 deaths in the United States, making it by far the most lethal cancer in men and women [41]. Our results have impact beyond a better understanding of cellular physiology and metabolism, with implications for the clinical management of malignant tumors of the lung. We not only show that ATG4B is high in patients with both LUAD and LUSC when compared to normal tissues, but that these elevations are found in early‐stage tumors, indicating that ATG4B may be promoting cancer progression through mTORC1 activation (a known driver of cancer pathology) early in the carcinogenic process. This idea is supported by the finding that ATG4B is not only associated with increased mortality, but with increased risk of cancer progression in LUAD, and that ATG4B gene expression remains high in metastatic tumors in both LUAD and LUSC. Further, ATG4B gene expression is higher in LUSC patients who smoked versus those who did not, and while differences between non‐smoking and smoking LUAD patients did not reach the level of statistical significance, ATG4B expression was higher in smokers versus reformed smokers, indicating that there may be an association between smoking and the pathological metabolic changes promoted by increased ATG4B expression. It is worth noting that ATG4B expression is higher in every category of smoking status (nonsmoker, current smoker, or reformed smoker) for both LUAD and LUSC, supporting its role as an oncogenic promoter of lung cancer pathology, regardless of the underlying risk factor of smoking.

However, cancer is in many ways a disease defined by heterogeneity, and lung cancer is no exception. Accordingly, we found differences between ATG4B expression patterns based on patient demographics and tumor characteristics. While there was no difference in ATG4B based on race in LUSC, we observed an increase in expression between Caucasian and African American patients in LUAD. These findings join others showing that the prevalence of oncogenic mutations is higher in African American patients with LUAD [42], with potential implications on disparities in cancer outcomes given our finding of an association between increased ATG4B expression and greater risk of mortality in LUAD patients. Finally, while ATG4B expression is not different in LUSC samples with or without a mutation in the tumor‐suppressing P53 genes (again higher than healthy tissues in both cases), we do report that ATG4B is higher in p53‐mutated LUAD tumors compared to both wild‐type cancers and healthy tissues. As studies have shown that mutation or deletion of the p53 gene is associated with increased autophagy [43, 44], this finding suggests that loss of the tumor suppression provided by p53 may allow for the shift toward rampant anabolic proteostasis observed in LUAD. Viewed as a whole, these results indicate that ATG4B is both a key component of altered lung cancer cellular anabolism via its relationship with mTORC1, and that ATG4B may be a driver of negative clinical outcomes in NSCLC.

Given the contribution of autophagy to cancer pathology, numerous efforts targeting this process as an anticancer therapy are underway. One of the most well‐studied autophagy inhibitors, chloroquine (and its derivative hydroxychloroquine), has long been shown to slow growth and enhance the effect of other therapies in lung cancer [45, 46]. However, while one meta‐analysis showed effectiveness with chloroquine‐based treatment combined with standard chemotherapy or radiation therapy [47], and hydroxychloroquine has proved to be well tolerated in a phase I trial of advanced NSCLC patients [48], studies in other cancer types have found limited efficacy and raised questions of tolerance of chloroquine/hydroxychloroquine administration. Accordingly, more select autophagy inhibitors have been developed, with several showing promise in preclinical studies. Given its centrality to the autophagic cascade, strategies targeting ATG4B have been reported in several cancer types, with anticancer effectiveness demonstrated in colorectal cancer [25], breast cancer [22], and osteosarcoma [49]. The compound utilized presently, NSC185058, is among several pharmacological inhibitors of ATG4B that have been successfully used to treat tumors, with the advantage that NSC185058 is relatively easily synthesized at scale. While true demonstration of ATG4B inhibitors as a clinically effective means of cancer treatment may still be on the horizon, our results, along with others, lay the groundwork for the use of this strategy in combating the growth of malignant tumors. It is worth noting that ATG4B is one of several isoforms in the ATG4 family. While the contributions of these various isoforms to cancer pathophysiology are still being elucidated [50], we elected to focus on ATG4B, which both has the highest affinity for the LC3B substrate [50] and has been well documented as being critical for cellular function across a range of cancers [51]. Future investigation into the relationship between these isoforms and mTORC1‐mediated anabolism is clearly warranted, as more research into the contributions of autophagic regulators to cellular anabolism will certainly result in a fuller view of the relationship between these regulatory pathways and their contributions to lung cancer.

Here we document that targeting ATG4B by either pharmacological inhibition or genetic knockdown halts cellular anabolism in NSCLC, resulting in reduced cell growth and suppressed protein synthesis rates. Further, we demonstrate that either route of targeting ATG4B leads to suppression of mTORC1 activity in a cellular model of LUAD, and that overexpression of ATG4B is sufficient to stimulate mTORC1 in healthy lung cells, indicating that ATG4B may drive the pathological changes in cellular signaling regulating NSCLC growth. As is evident from these results, the anabolic effects of mTORC1 and catabolic effects of the autophagic cascade are not independent, or even opposed, processes, but instead are fundamentally entwined to the point that mTORC1‐mediated protein synthesis cannot proceed without input from the autophagic pathway. In this way, we posit that autophagy (in this case mediated by ATG4B) is required for shifting cancer cell proteostasis to an anabolic mode, functioning as a vital input for mTORC1 activity. We argue that this represents a direct interaction between ATG4B‐regulated autophagy and mTORC1 and not one mediated by changes in energy balance, as combining either NSC185058 or si‐ATG4B treatments with the AMPK (a cellular energy sensor and known mTORC1 inhibitor) antagonist Compound C did not alter the effect of ATG4B targeting on cellular proliferation. These findings have implications outside of basic molecular biology, as we show that ATG4B gene expression is greater in NSCLC compared to healthy controls, is elevated in early stages of cancer development, and that high ATG4B levels are associated with increased risk of mortality and progression in LUAD. Thus, we propose that this novel interaction between ATG4B and mTORC1 represents not only a new insight into the fundamental mechanisms of cancer cell biology, but a viable avenue for effective precision anticancer therapy aimed at malignant tumors of the lung.

## Conflict of interest

The authors declare no conflict of interest.

## Author contributions

PJR and JDF designed and conducted the experiments and prepared the manuscript. BCG, SU, and MJG assisted with cell culture, western blot, and protein synthesis measurements. JMC procured the NSC185058 inhibitor and assisted in the preparation of the final manuscript. SER assisted in preparing the final manuscript and provided advice on statistical measures. All authors have read and approved the final manuscript.

## Supporting information


**Fig. S1.** NSC185058 and siATG4B reduce ATG4B protein content in NSCLC.
**Fig. S2.** AMPK does not mediate the effects of ATG4B on cell proliferation.
**Fig. S3.** ATG4B expression is not different between sexes in NSCLC. [Correction added on 25 February 2026, after first online publication: Fig S1 and Fig S2 has been updated].

## Data Availability

ATG4B gene expression was performed using the UALCAN platform, available at: https://ualcan.path.uab.edu/index.html. Survival analysis was performed using the Kaplan–Meier plotting tool, available at: https://kmplot.com/analysis/index.php?p=service&cancer=lung. All other data will be made available upon request to the corresponding author.
