# Supplementary material for: ATG4B is required for mTORC1‐mediated anabolic activity and is associated with clinical outcomes in non‐small cell lung cancer
Source: FEBS Open Bio. 2025 Oct 9;16(3):570–83. doi: 10.1002/2211-5463.70138 (PMC12955743; doi:10.1002/2211-5463.70138)
Supplement: Supplementary file 1 — Fig. S1. NSC185058 and siATG4B reduce ATG4B protein content in NSCLC. Fig. S2. AMPK does not mediate the effects of ATG4B on cell proliferation. Fig. S3. ATG4B expression is not different between sexes in NSCLC. [Correction added on 25 February 2026, after first online publication: Fig S1 and Fig S2 has been updated]. [file FEB4-16-570-s001.pdf]

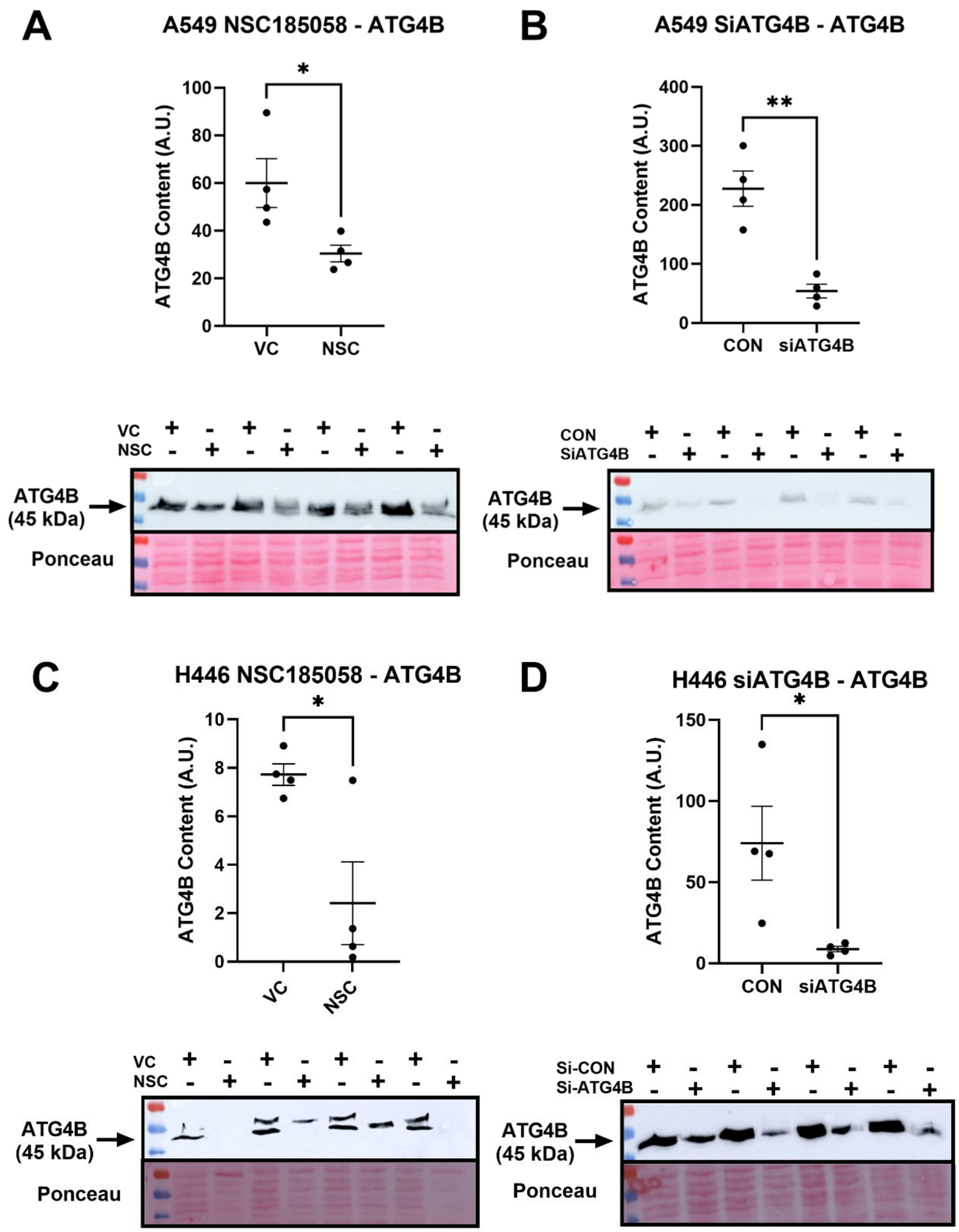

Supplemental Figure 1: NSC185058 and siATG4B reduce ATG4B protein content in NSCLC. Both pharmacological treatment (NSC185058) and silencing RNA (siATG4B targeting ATG4B cause reductions in ATG4B protein content in lung adenocarcinoma (LUAD, panels A-

D) and squamous cell carcinoma (LUSC, H446 cell line, panels E-H). * indicates a significant difference ($p<0.05$), ** ($p<0.01$), *** ($p<0.001$), **** ($p<0.0001$), assessed by two-way t-test. VC = vehicle control, NSC = NSC18505. Figures are presented as means with standard errors, all n = 4.

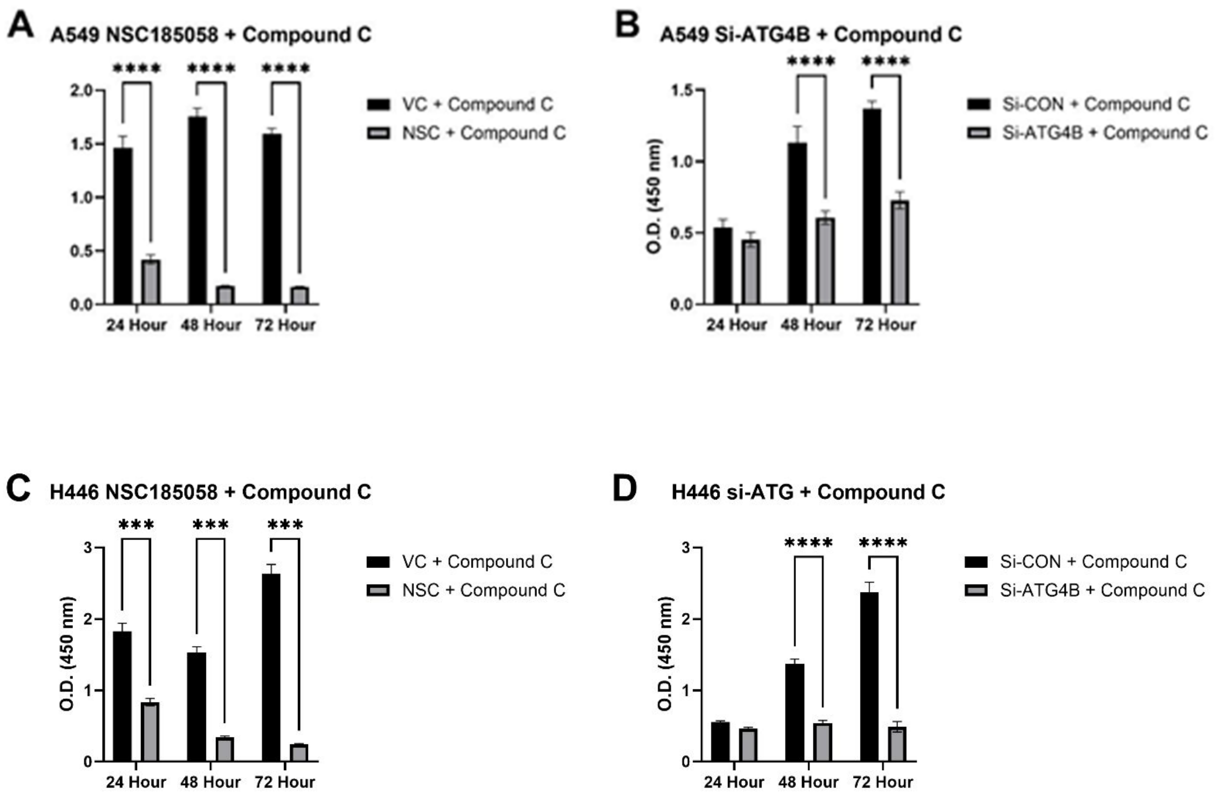

Supplemental Figure 2: AMPK does not mediate the effects of ATG4B on cell proliferation. Repeating the proliferation assays described in Figure 1 in the presence of the AMPK inhibitor Compound C (dorsomorphin) does not alter the effects of ATG4B targeting, indicating that AMPK is not a mediator of the contributions of ATG4B to cell signaling. Statistical significance was assessed by two-way ANOVA with a Sidak correction in the case of significant differences Figures are presented as means and standard errors, all n = 4. * indicates a significant difference (p<0.05), ** (p<0.01), *** (p<0.001), **** (p<0.0001), assessed by two-way ANOVA with a Sidak correction in the case of significant differences. VC = vehicle control, NSC = NSC18505. Figures are presented as means with standard errors, all n = 4.

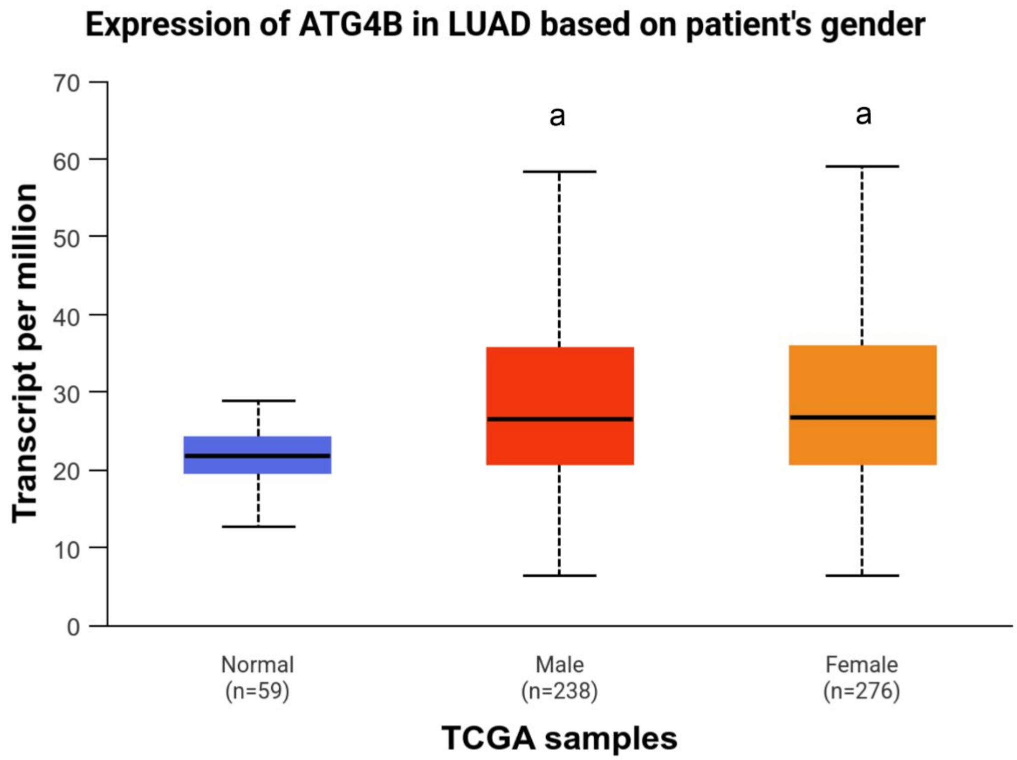
Expression of ATG4B in LUAD based on patient's gender
Transcript per million
0
10
20
30
40
50
60
70
a
a
Normal (n=59)
Male (n=238)
Female (n=276)
TCGA samples

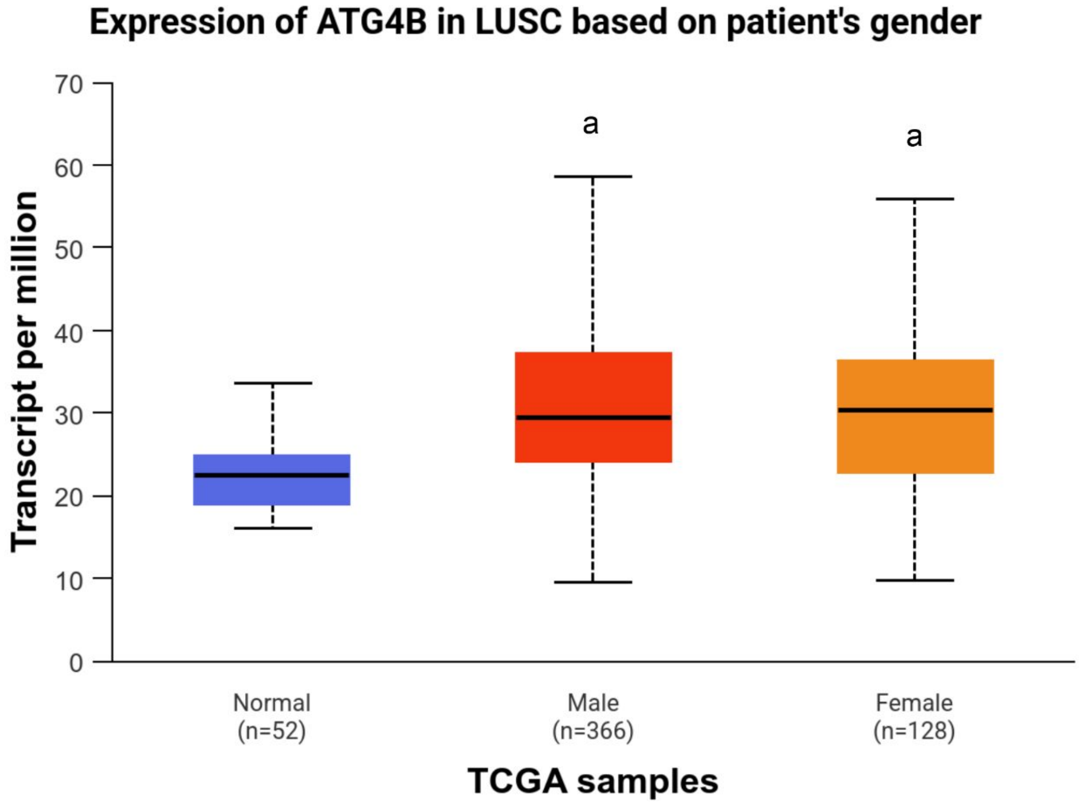
Expression of ATG4B in LUSC based on patient's gender
Transcript per million
0
10
20
30
40
50
60
70
a
a
Normal (n=52)
Male (n=366)
Female (n=128)
TCGA samples

Supplemental Figure 3: ATG4B expression is not different between sexes in NSCLC.

ATG4B gene expression is elevated compared to normal tissues in patients of both sexes in both lung adenocarcinoma (LUAD) and lung squamous cell carcinoma (LUSC). Figures are presented as box and whisker plots with respective n listed below the x-axis. Statistical significance was assessed by Welch's test, "a" indicates a significant difference ($p<0.05$) from normal tissues.
